# Optical properties of functionalized nanodiamonds

**DOI:** 10.1038/s41598-017-14553-z

**Published:** 2017-10-26

**Authors:** V. Pichot, O. Muller, A. Seve, A. Yvon, L. Merlat, D. Spitzer

**Affiliations:** 1 NS3E “Nanomatériaux pour Systèmes Sous Sollicitations Extrêmes” UMR 3208 ISL/CNRS/UNISTRA, French-German Research Insitute of Saint-Louis, 5 rue du général Cassagnou, 68301 Saint-Louis, France; 2 Radiation Interaction with Matter Laboratory, French-German Research Insitute of Saint-Louis, 5 rue du Général Cassagnou, 68301 Saint-Louis cedex, France

## Abstract

Detonation nanodiamonds exhibit strong nonlinear optical properties depending on their electronic properties. In the present paper, the nanodiamond functional groups are chemically modified to obtain nanodiamonds with primary amines on their surface. The optical properties of such nanodiamonds placed in water suspensions are studied and compared with the one of classical detonation nanodiamonds. Transmission, scattering and Z-scan experiments are performed for two different wavelengths (532 nm and 1064 nm). A lower threshold for optical limiting associated to more pronounce non-linear optical effects is detected at the wavelength of 1064 nm compared to the one at 532 nm. This effect may be due to a stronger nonlinear backscattering behavior at 1064 nm. Moreover, a striking result obtained from the Z-scan experiments reveals a completely different behavior of the functionalized nanodiamonds for both wavelengths. This result is discussed in regard to the electronic properties of the material and possible charge transfer.

## Introduction

With the nanomaterial advent, the behavior of nanoparticles when submitted to an electromagnetic solicitation has been intensively studied. Among nanoparticles which are of great interest in the optical field, carbonaceous nanoparticles have demonstrated extremely interesting properties in order to develop nonlinear optical systems^1,2^. Moreover, previous studies showed that well controlled detonation nanodiamonds (DNDs) suspensions or functionalized DNDs could greatly improve the nonlinear properties of such material^3,4^.

DND is a material of great interest due to its small size, the numerous functional groups present at its surface and its electronic properties. The variety of functional groups that can be found on the DND surface is depending on the purification or post purification treatments. In this manner, oxygenated, hydrogenated, chlorinated groups can be found on the DND surface^5–10^.

In previous studies, Schmidlin *et al*. showed the presence of hydroxyl and carboxylic acid groups on DNDs purified under air treatment^5^. While carboxylic acids where mainly located on the most reactive parts of the particles most of the surface was covered with hydroxyl groups. The work of Shenderova *et al*. is in good agreement with these results^11^. Research groups modified the DNDs surface functional groups into hydrogenated ones by using plasma or heat treatment under hydrogen flow in order to produce individual DND suspensions or to use them for functionalization or medical applications^6,8,12^. Krüger *et al*. showed wide possibilities to functionalize the diamond with several functional groups and molecules^10^.

Varying the functional groups may change the electronic properties of the material and therefore change their optical properties^13^. Sasagawa *et al*. showed by using ultraviolet photon spectroscopy that the Fermi level of hydrogenated diamond is modified compared to the one of oxygenated nanodiamonds.

The goal of this study is to obtain nanoparticles with nonlinear properties to serve as optical filters to limit the effect of aggressive laser toward optronic systems (sensors, eyes…). DNDs can be used in suspension in appropriate solvent or in composites material such as polymers. For this purpose, functionalization can be necessary in order to maintain the DNDs in colloidal suspensions or for further integration in a solid matrix. Aminated DNDs seem very interesting for their possible interaction with hydroxyl groups of some polymers.

In this article, we describe the functionalization of DNDs with NH_2_ groups on their surface by using a (S)-*N*-Boc-2,3-epoxypropylamine molecule. The optical properties of the modified diamonds are investigated in the green and near infrared region. The transmittance, scattering and z scan experiments are performed and reveal striking results for this complex.

## Results and Discussion

### DND functionalization

**Figure 1:**
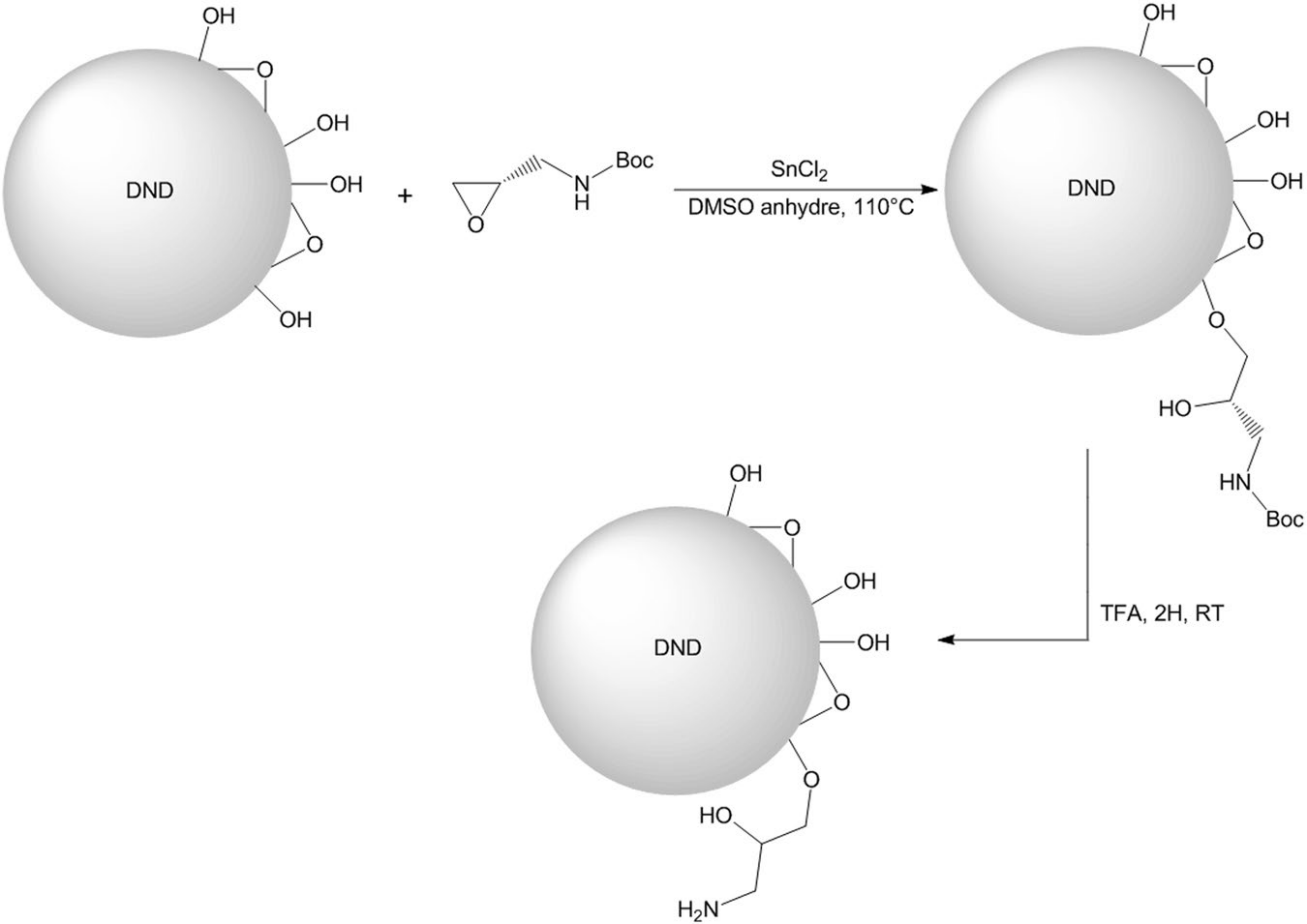
Reactional scheme of the DND functionalization. Functionalization of the nanodiamond surface with the (S)-*N*-Boc-2,3-epoxypropylamine molecule and deprotection of the amine group.

The hydroxyl groups present at the DNDs surface were used in order to functionalize them. A DND particle with a diameter of 5 nm exhibits around 800 surface atoms. The quantity of (S)-*N*-Boc-2,3-epoxypropylamine used in the reaction was adjusted in order to graft around 100 of this molecule on the DND surface. The epoxy group of the (S)-*N*-Boc-2,3-epoxypropylamine molecule was used to form covalent bond with the OH groups^14^. The deprotection of the amine group was achieved to obtain the primary amine group. The reactional scheme is given in Fig. 1.

Different techniques (infrared and Raman spectroscopies, colorimetric tests) were used in order to prove the presence of the (S)-*N*-Boc-2,3-epoxypropylamine at the surface of the DND. These characterizations were unsuccessful probably due to the weak yield of this reaction.

A dye molecule (porphyrin) that can only be grafted on NH_2_ groups was grafted on the functionalized DND in order to demonstrate the efficiency of the grafting with the (S)-*N*-Boc-2,3-epoxypropylamine molecule. This reaction was achieved by activation the carboxylic group of the (5-4-carboxyphenyl)-10,15,20-triphenyl-21,23-porphyrin with Hydroxybenzotriazole (HOBt) for the racemisation and Dicyclohexylcarbodiimide (DCC). This reaction was performed separately on both raw DNDs and DNDs grafted with deprotected (S)-*N*-Boc-2,3-epoxypropylamine (DND-NH_2_). If one of these two samples (DND porphyrin and DND-NH_2_ porphyrin) possess some primary amines groups, the porphyrin should be grafted at its surface after this reaction.

**Figure 2:**
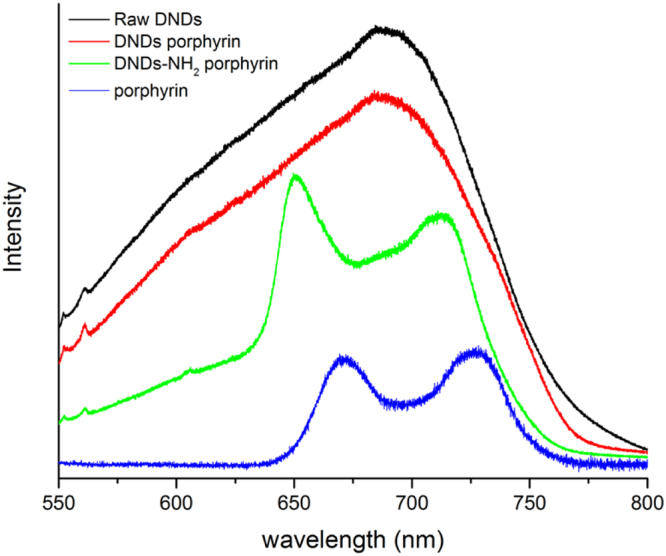
Photoluminescence spectra of the samples. Photoluminescence Raman Spectra obtained for DND (black), DND porphyrin (red), DND-NH_2_ porphyrin (green) and porphyrin alone (blue). A 514 nm excitation wavelength was used.

The samples were rinsed several time after the reaction with Dymethyl Sulfoxide (DMSO). DMSO is a very good solvent for this porphyrin. Finally, water was used to remove the remaining DMSO. The photoluminescence of the resulting DNDs was analysed by using the Raman spectrophotometer. The recorded spectra are given in the Fig. 2.

The spectra obtained from the samples containing DNDs show the signal from the DND at 552 nm and 561 nm corresponding to the diamond lattice vibrations band and to amorphous carbon or interstitial carbon atoms in the bulk of the crystallites respectively^15^. While the one of DND porphyrin is identical to the one of raw DND the spectrum of DND-NH_2_ porphyrin reveals some additional photoluminescence bands at 650 and 713 nm. These bands are typical of the (5-4-carboxyphenyl)-10,15,20-triphenyl-21,23-porphyrin as shown from the spectrum of the porphyrin alone (blue curve). The blue shift of these photoluminescence bands can be attributed to interactions between the DNDs and the molecule due to the grafting of the porphyrin on the DNDs surface.

According to this result, we can clearly assume the successful grafting of the (S)-*N*-Boc-2,3-epoxypropylamine on the DND surface and the removal of the tert-Butyloxycarbonyl (Boc) protecting group function leading to the presence of NH_2_ pending bond on the DND.

Nitrogen is present inside the DNDs crystals as defaults with yield of around 1–2 at.%. The variation of the nitrogen can vary by 1 at.% from one nanodiamond sample to another making quantitative studies such as XPS or elementary analyses very difficult to interpret. However, X-Ray Photoelectron Spectroscopy (XPS) measurements have been performed on DND and DND-NH_2_ samples, the results are given in Table 1. No difference between the two samples could be noticed. Remaining Tin (Sn) coming from the SnCl_2_ catalyst used in the reaction was detected in the DND-NH_2_ sample. This means that even with several washing of the sample some Tin is still present in this sample.

**Table 1:** XPS quantitative measurements on DND and DND-NH_2_ from C1s, O1s, N1s and Sn3d signals.

Samples	signal	Position	At.%	St.Dev. %
DND	C 1 s	285,00	87,46	0,28
	O 1 s	531,00	10,75	0,14
	N 1 s	399,00	1,80	0,28
DND-NH_2_	C 1 s	285,00	85,79	0,31
	O 1 s	530,50	11,63	0,16
	N 1 s	398,50	1,83	0,30
	Sn 3d	485,00	0,74	0,05

**Figure 3:**
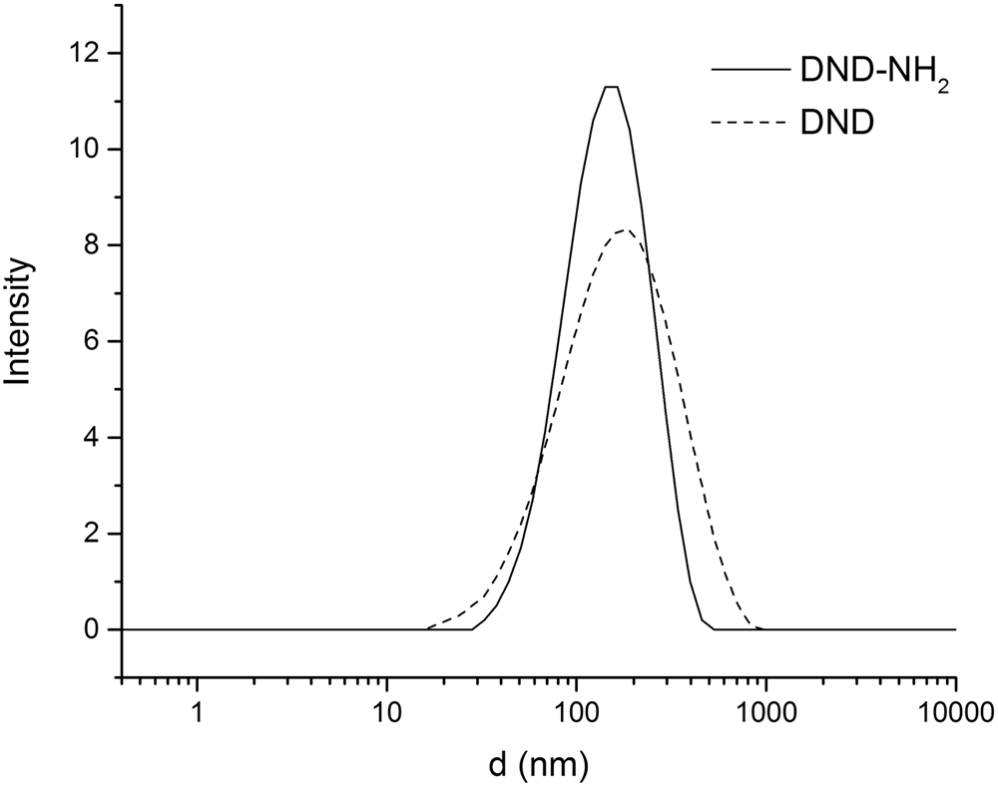
Size analyses of the DND samples. Dynamic light scattering of the raw DND and DND-NH_2_.

The analysis of DND-NH_2_ suspension performed by Dynamic Light Scattering (DLS) reveals a size distribution around 120 nm for the DND aggregates (Fig. 3). This size distribution is similar to the one obtained for the raw DND suspension and is classical for suspensions obtained from supernatant after 1 hour of ultrasound solicitation and one day of sedimentation^3^. This means that the functionalization of the DNDs into DND-NH_2_ did not lead to aggregation of the nanodiamonds as the concentration of the supernatant are identical.

### Nonlinear transmittance

**Figure 4:**
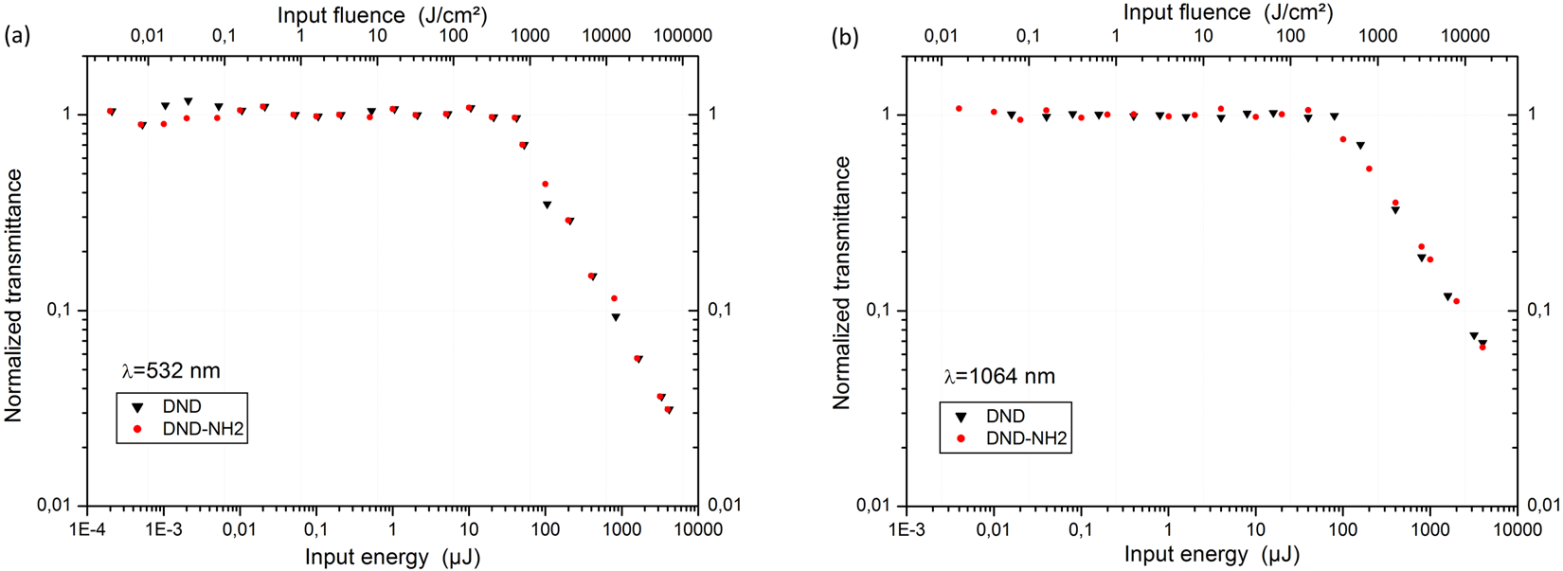
Non-linear transmittance experiments. Normalized transmittance as a function of the input energy and input fluence in a log-log scale. DND and DND-NH_2_ suspensions in water at λ = 532 nm (**a**) and λ = 1064 nm (**b**) are represented.

Figure 4 shows the normalized nonlinear transmittances at both laser wavelengths (532 nm and 1064 nm) for the DND and DND-NH_2_ suspensions in water. The input fluence was set to vary from 1 mJ/cm^2^ to approximately 10^5^ J/cm^2^. The observed behaviors for both nanomaterials are very similar. The energy or fluence thresholds for optical limiting assessed in such a way where the transmittance drops to 50% are the following at wavelengths of 532 nm and 1064 nm, respectively: 1200 J/cm^2^ and 870 J/cm^2^. At 532 nm, a nonlinear attenuation corresponding to an optical density of 1.5 is achieved for both the DND and DND-NH_2_ nanomaterials. At 1064 nm the trend remains approximately unchanged and an optical density of 1.2 is reached. In our experiments at 1064 nm (Fig. 4b) the transmittance drop begins at a slightly lower input fluence in comparison with the one at 532 nm (Fig. 4a). It is an interesting observation since the largest decrease in transmittance is obtained at 532 nm. This result is in contradiction with a previous study on metallic nanoparticles^16^. Accordingly, it is worth noting that the low threshold for optical limiting will be associated to more pronounced nonlinear optical effects at the wavelength of 1064 nm in the range 500 J/cm^2^ to 1500 J/cm^2^. Our postulate will be confirmed in a next section of this present work.

### Nonlinear scattering

**Figure 5:**
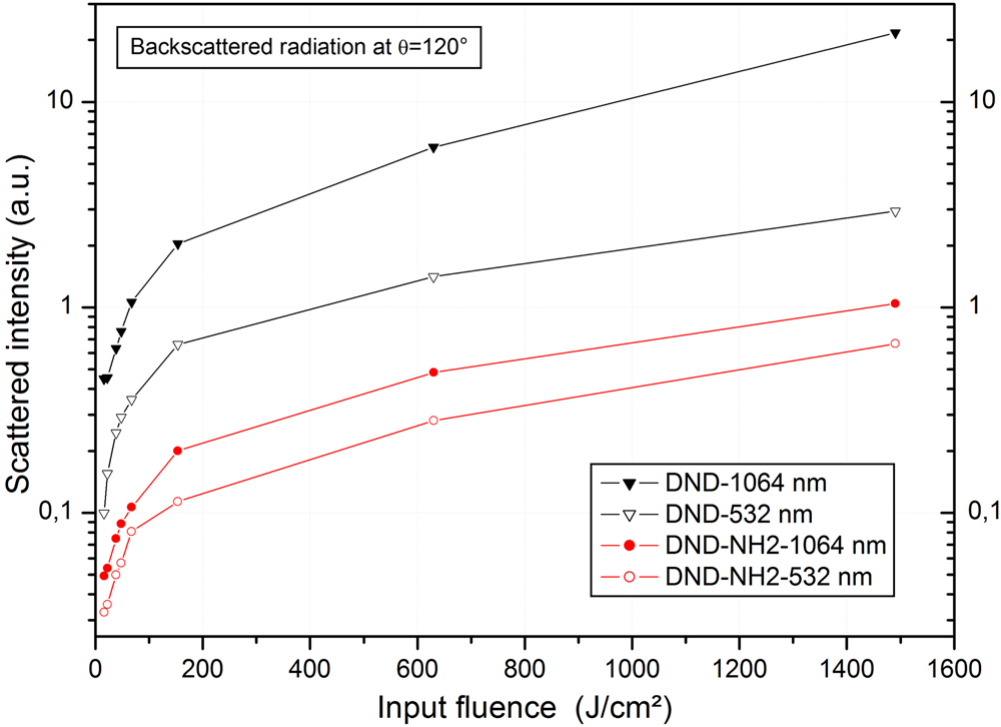
Non-linear scattering experiments. Semilog plot of the backscattered intensity (θ = 120 degree) as a function of the incident fluence at λ = 532 nm (open symbols) and λ = 1064 nm (filled symbols) for the DND and DND-NH_2_ suspensions.

To better understand the reason that accounts for this threshold difference, nonlinear scattering measurements were conducted on DND and DND-NH_2_ at both laser wavelengths. Generally speaking, after the laser has impacted the suspension, scattered waves are generated and propagate in all directions defined in a polar diagram^17^. It is obvious to say that only the backscattered waves contribute to the optical limiting phenomenon since these latter are rejected in a direction opposite to the sensor or the optronic device to be protected. In this way, we assessed the nonlinear backscattering properties of the DND and DND-NH_2_ systems at an angle of 120 degree. The results are shown on Fig. 5 giving the scattered intensity as a function of the incident fluence in the range of interest.

It can be clearly seen that the scattered intensity is larger at a wavelength of 1064 nm for both nanomaterials especially for input fluencies above 600 J/cm^2^. Figure 5 shows that the backscattered radiation is about a factor 10 more intense at 1064 nm compared to 532 nm in the case of the DND. However, this difference is less significant for the DND-NH_2_. The strong nonlinear backscattering behavior at 1064 nm detected in the fluence range [800–1500] J/cm^2^ could explain a lower value for the onset of nonlinearities at 1064 nm as observed on the Fig. 4. Besides nonlinear scattering one can expect the occurrence of other optical nonlinearities like nonlinear absorption and nonlinear refraction^18^, or the electronic Kerr effect^19^. Our Z-scan measurements performed in a close aperture scheme will help to discriminate whether one of the aforementioned nonlinearity occurs in the DND and DND-NH_2_ nanomaterials systems.

### Z-scan experiments

**Figure 6:**
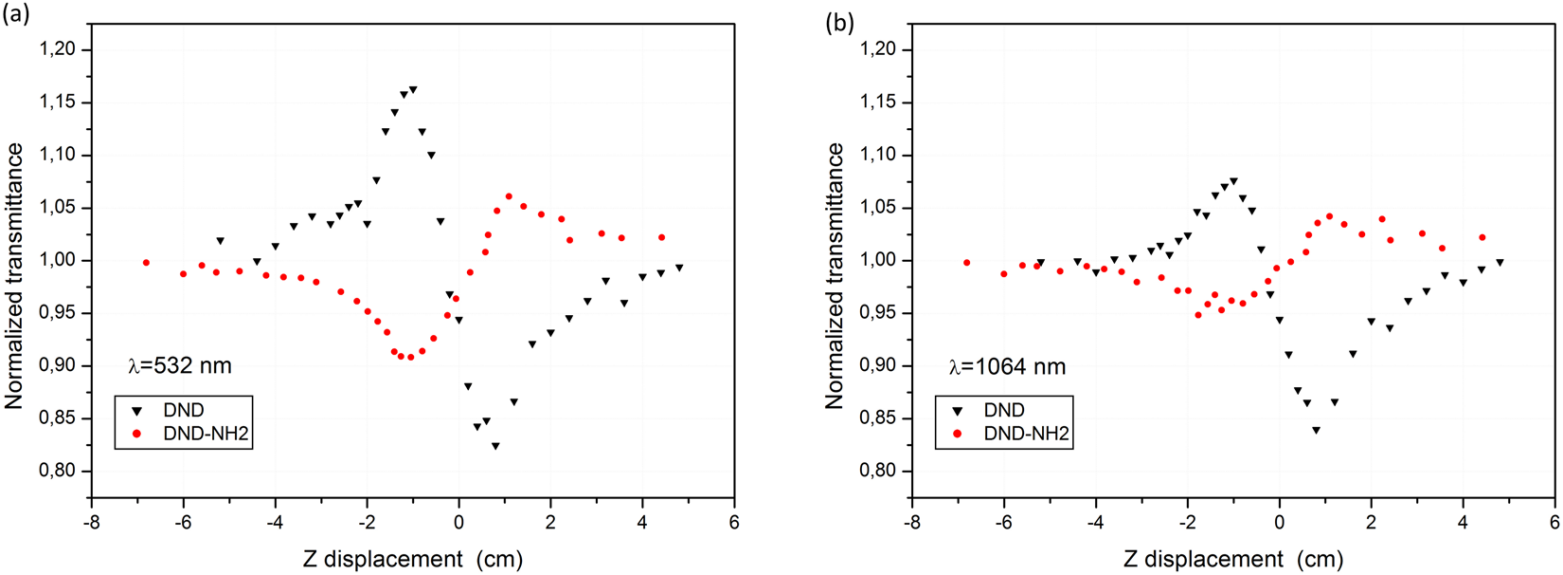
Z-scan experiments. Close Z-scan signatures of the DND and DND-NH_2_ suspensions for an incident fluence of F = 1000 J/cm^2^. λ = 532 nm (**a**) and λ = 1064 nm (**b**). The curves reveal as well a self-defocusing (DND) and a self-focusing effect (DND-NH_2_).

The results are given in Fig. 6 reporting the normalized transmittance as a function of the Z sample displacement.

It is to be pointed out that symmetric Z-scan transmittance signatures are obtained at λ = 532 nm for both nanomaterials revealing that nonlinear absorption does not contribute to the observed phenomenon^18^ (Fig. 6a). Indeed, in the case of DND the peak to valley shape indicates a negative nonlinear refraction corresponding to a negative sign of the real part of the third order susceptibility^20^ (Reχ^(3)^ > 0). From a previous work of O. Muller *et al*.^16^, it is to be pointed out that such a result was expected since nanosecond duration laser pulses (this work, 4 ns) are mainly responsible for thermal lenses induced nonlinearities. Using the experimental results in Fig. 6 together with the analytical solution of the well-known Z-scan formalism detailed in^16^, the nonlinear refractive index, *n*_2_, of the DND was calculated to be *n*_2_ = −2.86·10^−16^ cm^2^/W which denotes weak nonlinearities.

On the other hand, the valley to peak shape recorded in the case of the DND-NH_2_ is somehow more surprising. Such a signature is subsequent to a self-focusing of the laser beam in the nonlinear sample, meaning, reversely to the former case, a positive sign of the real part of the third order susceptibility (Reχ^(3)^ > 0). Generally speaking, such a behavior is observed when nonlinear materials are submitted to ultra-short laser pulses (hundreds of picoseconds and less) and is currently attributed to Kerr effects nonlinearities.

The DND-NH_2_ sample contains NH_2_ functional groups and remaining catalyst (Tin). Additional Z-scan experiments were performed on two complementary samples: raw DND mixed with SnCl2 in water for one night at 110 °C and raw DND mixed with SnCl2 in water for one night at 110 °C followed by a treatment with TFA the next day. For both samples, it was found the same peak-valley behavior like raw DNDs sample (not shown here). This means that the NH_2_ functional groups is responsible of the valley to peak shape recorded in the case of the DND-NH_2_.

Assuming that intra and intermolecular charges transfer occur most likely in the DND-NH_2_, the sign change of the nonlinear refractive index could be explained. However, such nonlinear phenomena take place on a picosecond timescale, which cannot be compared to the duration of the laser pulses of the present study. Accordingly, it might be possible that a tenfold occurrence of ultra short nonlinearities where molecular reorientational Kerr effect cumulates with enhanced charges transfer could be responsible for the observed valley to peak shape. Indeed, the NH_2_ moiety could alternatively act as an electron donor (by conjugation) when the N atom, through its free electron pairs, participates to the resonance, or as a withdrawing group if the N atom cannot participate to the conjugation.

The same behavior is observed at λ = 1064 nm (Fig. 6b) and a value of *n*_2_ = −2.34.10^−16^ cm^2^/W is deduced for the DND, similar to the one assessed at λ = 532 nm. However, the valley to peak shape shown for the DND-NH_2_ is less pronounced in comparison to the case λ = 532 nm, thus a precise analytical calculation of the nonlinear refractive index could not be performed.

## Conclusions

We have shown that chemically modified DNDs can exhibit a very different behavior to laser solicitation compared to non-modified DNDs. While for both samples a more pronounced nonlinear backscattering behavior is observed at 1064 nm, the DND-NH_2_ revealed a valley to peak shape in the Z-scan experiments, which can be attributed to Kerr nonlinearities effects. This very important result shows that it is possible to strongly influence the optical properties of the detonation nanodiamonds. Further studies are needed to better explain the origin of these phenomena especially by investigating the electronic properties of such materials.

## Methods

Nanodiamonds were synthesized at the French German research institute of Saint-Louis (ISL) by detonation of high explosive mixtures (hexogen and trinitrotoluene) in a detonation chamber. After the explosion the detonation soot containing the DNDs is recovered by rinsing the detonation tank with deionized water. The DNDs purification is performed in two steps: an acid washing with a mixture of HCl/HNO_3_ followed by an oxidation of the resulting soot at 380 °C.

The molecule used for the nanodiamond functionalization is (S)-*N*-Boc-2,3-epoxypropylamine (sigma aldrich 97%). Nanodiamonds (500 mg) were put together with (S)-*N*-Boc-2,3-epoxypropylamine (70 mg), SnCl_2_ (70 mg) was added to serve as catalyst, the reaction was performed at 110 °C during 24 h in water (100 mL) under reflux with a magnetic stirring. After the reaction, the resulting product was rinsed several times with water and dimethylsuloxyde (DMSO sigma Aldrich, 99+%) over a 1 µm membrane filter (Millipore) to remove the (S)-*N*-Boc-2,3-epoxypropylamine excess. trifluoroacetate acid (14 mg, sigma Aldrich 99+%) and water (100 mL) were added at room temperature during 2 h under magnetic stirring to remove the tert-Butyloxycarbonyl protecting group (BOC group). The final product was then once again rinsed with water over a 1 µm membrane filter (Millipore) until obtaining a neutral pH.

A suspension of modified nanodiamonds was prepared in deionized water with a concentration of 1 g/L. After sedimentation of the biggest aggregates during 24 h, the stable supernatant phase was recovered and used for optical experiments.

**Figure 7:**
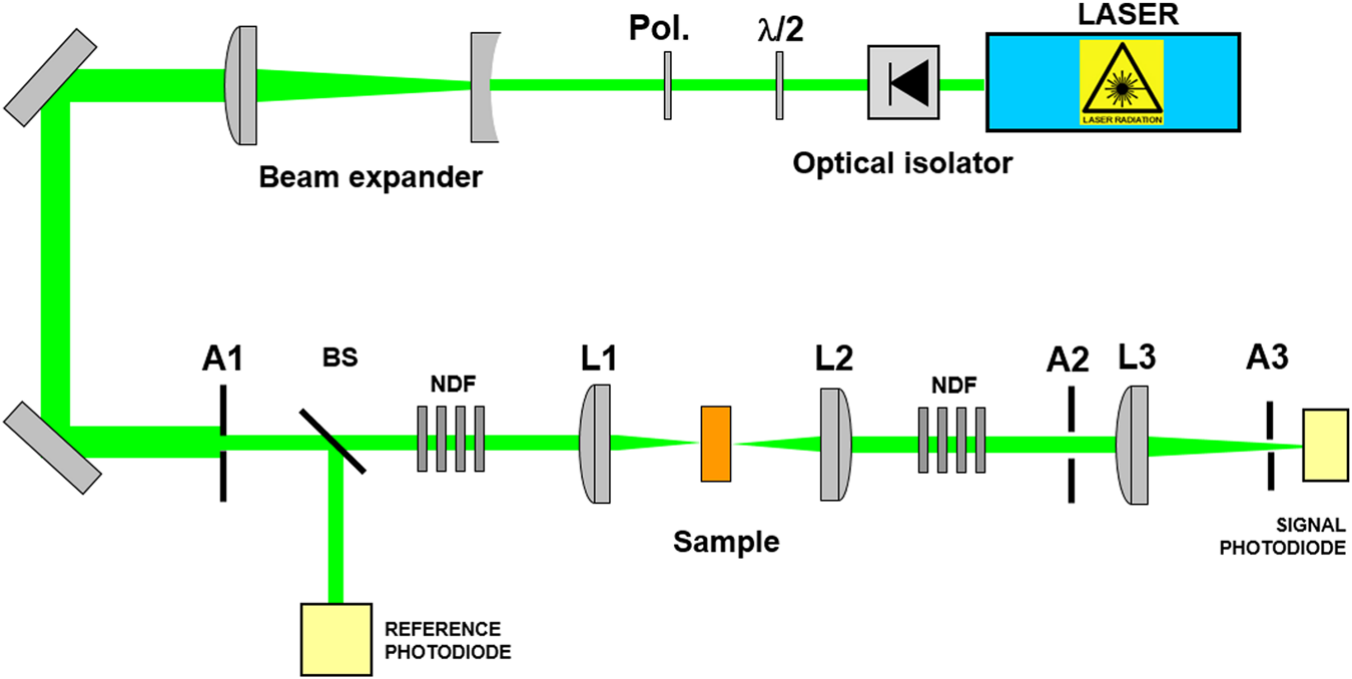
Optical experimental setup. Experimental setup used to study the optical limiting behavior of nonlinear nanomaterials samples. The denoted components are explained in the text.

A frequency doubled Nd:YAG laser system (Quantel) is used as the laser source, its output energy extents up to 400 mJ at 1064 nm and 160 mJ at 532 nm. The pulse repetition rate was fixed to 1 Hz and the pulse width is 4 ns. The experimental setup used to assess the nonlinear transmittance is shown in Fig. 7. The entrance aperture A1 was overfilled by the expanded beam so that a top-hat spatial irradiance distributed beam resulted. The nanomaterials samples were placed at the intermediate focal plane of a Keplerian telescope made of lens L1 and L2 with focal lengths of 60 mm and 100 mm, respectively. In front of the input lens L1 and behind the output lens L2, the apertures A1 and A2 (12 mm and 20 mm, respectively) were placed to achieve an optical system with a f-number of f = 5. With the help of a beam profiler (Cohu CCD camera), the focal diameter was estimated to be 4 µm at 532 nm and 8 µm at 1064 nm. A part of the laser beam is splitted off by the beam splitter BS to monitor the incident energy, whereas the laser beam transmitted through the sample is further focused using the lens L3 (focal length 400 mm) to measure the signal energy. Additionally, the aperture A3 with an opening diameter of 600 µm is positioned at the focal point of L3 toward the signal photodiode.

**Figure 8:**
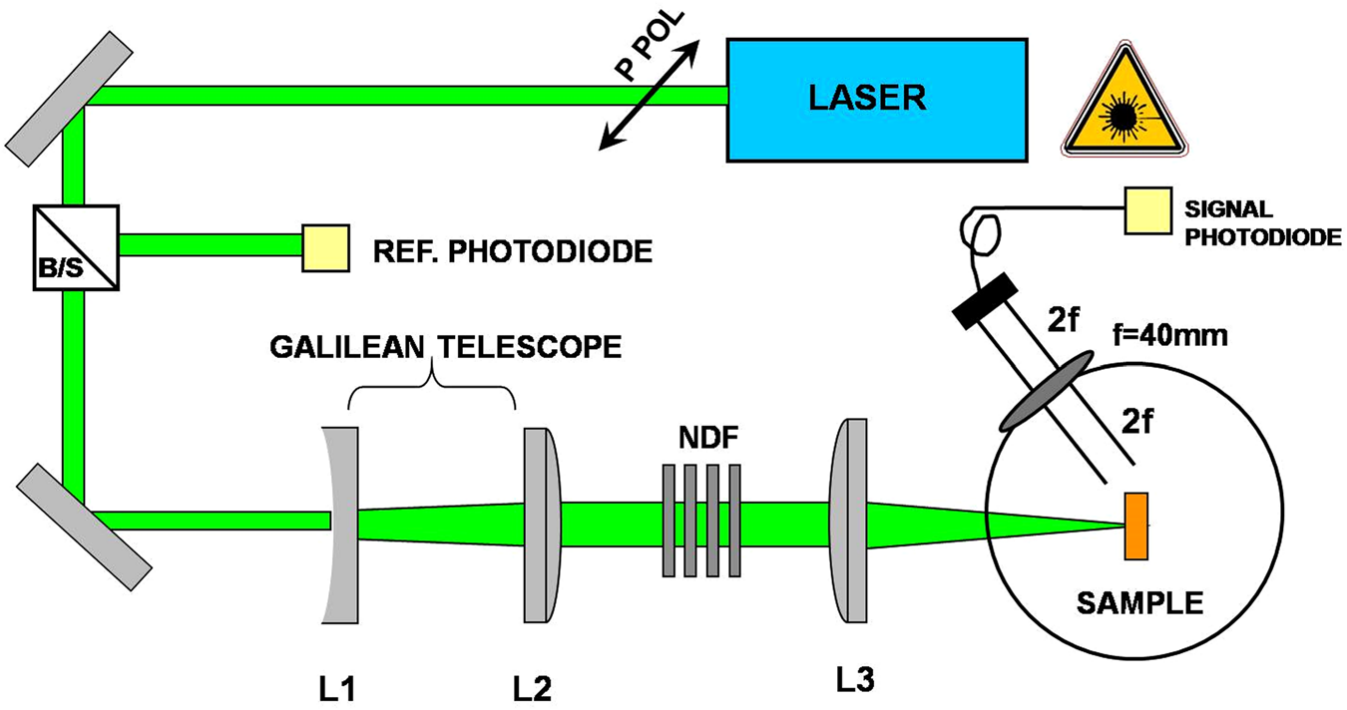
Experimental setup used to study the polar scattering properties. Details are in the text.

The setup used for the measurement of the polar nonlinear scattering is given on Fig. 8. Experimental details on the setup are reported elsewhere^21^. Shortly, the beam first expanded is focalized by the lens L3, f3 = 200 mm. The sample is set in the focal plane of L3 and the radius of the laser beam at the focus is 30 µm. The scattered signal is recorded on a photodiode mounted on a rotating stage used to collect the scattered light at an angle of 160 degree related to backscattering.

**Figure 9:**
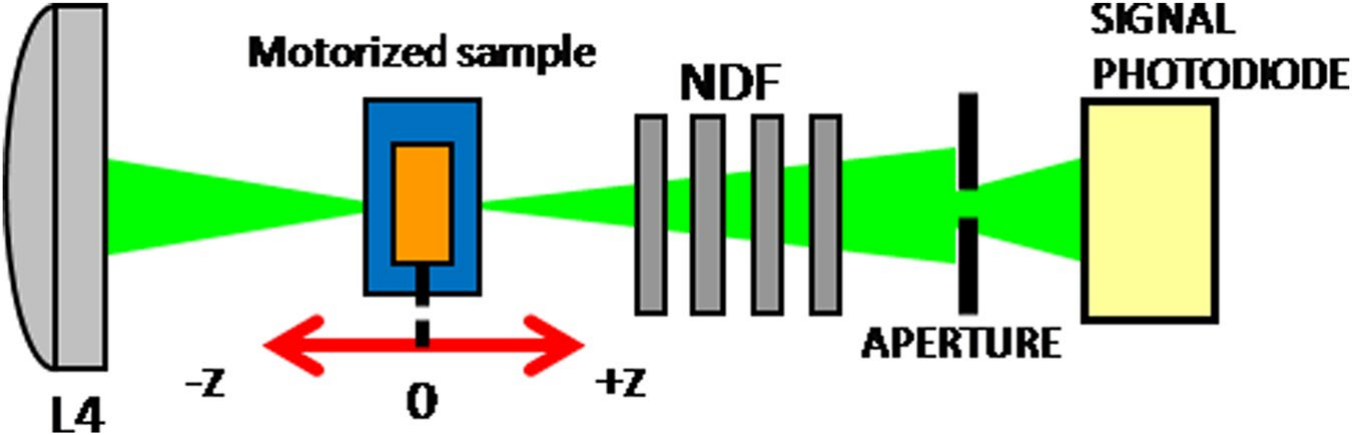
Z-scan experimental apparatus. The denoted components are explained in the text.

One part of the incident laser beam is taken from the main setup (Fig. 9) and directed toward the Z-scan setup in its close aperture scheme as shown in Fig. 2. The incident beam with a diameter of 7 mm is focused into the sample area through the lens L4, f4 = 200 mm in a f/30 focusing geometry^4^. The sample is placed on a motorized stage and is moved from −z to +z. The emerging signal is collected on a photodiode before which we placed a 600 µm aperture. The use of 1 mm thin cuvettes follows the statement that the medium is to be considered as thin, i.e. a thickness smaller than the diffraction length of the focused beam^16^.

Raman spectra were recorded by using invia Renishaw spectrometer with a 514 nm laser in photoluminescence mode including an edge filter and a grating of 1800 l/mm. Data were collected by a CCD camera.

Dynamic Light Scattering (DLS) was performed on a Malvern Instruments Zetasizer to measure the size of the nanodiamonds aggregates in suspension in water.

X-Ray Photoelectron Spectroscopy was achieved at the “Institut de Sciences des Matériaux de Mulhouse” (IS2M) with a VG SCIENTA “SES-2002” spectrometer, the samples were prepared on double sided adhesive carbon conductive tape. A monochromatic Al Kα_1,2_ X-Ray source was used.
